# Natural tuning of restriction endonuclease synthesis by cluster of rare arginine codons

**DOI:** 10.1038/s41598-019-42311-w

**Published:** 2019-04-09

**Authors:** Iwona Mruk, Tadeusz Kaczorowski, Agata Witczak

**Affiliations:** 1 Department of Microbiology, Faculty of Biology, University of Gdansk, Wita Stwosza 59, Gdansk, 80-308 Poland; 2 Laboratory of Extremophiles Biology, Department of Microbiology, Faculty of Biology, University of Gdańsk, Wita Stwosza 59, Gdansk, 80-308 Poland

## Abstract

Restriction–modification (R-M) systems are highly widespread among bacteria and archaea, and they appear to play a pivotal role in modulating horizontal gene transfer, as well as in protecting the host organism against viruses and other invasive DNA particles. Type II R-M systems specify two independent enzymes: a restriction endonuclease (REase) and protective DNA methyltransferase (MTase). If the cell is to survive, the counteracting activities as toxin and antitoxin, must be finely balanced *in vivo*. The molecular basis of this regulatory process remains unclear and current searches for regulatory elements in R-M modules are focused mainly at the transcription step. In this report, we show new aspects of REase control that are linked to translation. We used the EcoVIII R-M system as a model. Both, the REase and MTase genes for this R-M system contain an unusually high number of rare arginine codons (AGA and AGG) when compared to the rest of the *E. coli* K-12 genome. Clusters of these codons near the N-terminus of the REase greatly affect the translational efficiency. Changing these to higher frequency codons for *E. coli* (CGC) improves the REase synthesis, making the R-M system more potent to defend its host against bacteriophages. However, this improved efficiency in synthesis reduces host fitness due to increased autorestriction. We hypothesize that expression of the endonuclease gene can be modulated depending on the host genetic context and we propose a novel post-transcriptional mode of R–M system regulation that alleviates the potential lethal action of the restriction enzyme.

## Introduction

Restriction-modification (R-M) systems are highly widespread among prokaryotes^1–3^. The most well-studied Type II R-M systems are composed of two independent enzymes: a restriction endonuclease (REase) and a DNA methyltransferase (MTase). Both proteins recognize the same short specific DNA sequence, but differ in function. The REase cleaves at the recognition sequence while the MTase adds methyl group to modify such sites, to make them protected from the action of the cognate REase^4^. This way the genomic DNA is marked as the self, in contrast to any foreign DNA entering the cell that lacks such modification (non-self) and become easily the target for degradation by host REase. Since their discovery, R-M systems have been recognized as the potent tools to combat bacteriophages. Along with CRISPR systems, they form the active line of bacterial cell defence^5–7^. By limiting the DNA flux into host cells, Type II R-M systems function as modulators of horizontal gene transfer and such may contribute to bacterial speciation^8–11^. Due to the potent endonucleolytic activity of REase, the R-M systems might also be considered as potentially hazardous cargo for their hosts. If the expression of REase and MTase is not balanced, then the host death is likely outcome^12–14^. The challenge then for R-M and CRISPR like systems is one of fine tuning the regulatory control over toxic genes in a manner that promotes high anti-phage immunity while minimizing self-destruction^15–17^. For mobilizable R-M genes^11,18^, tight control over REase is especially important at the stage of transfer to a new host cell. At this stage the genome has not been protected by MTase and is susceptible to degradation. R-M systems must have a time-dependent control that initially favours the expression and action of the MTase if the newly introduced gene are to be acquired by the new host^19–22^. The details underlying these molecular processes are unclear, though it seems that genetic feedback loops play a central role. In this case of R-M gene expression regulation has been studied more extensively for fixed genes, at steady-state. In general, three modes^23^ of gene control for Type II R-M systems are in use: regulatory MTase^24–26^; antisense RNA^27,28^; and a dedicated transcription factor called the C protein^29–34^. Co-regulatory modes are very likely to be discovered.

Thus far, studies of the regulatory elements involved in Type II R-M system gene expression have been focused at the stage of transcription, and to our knowledge, no report has yet revealed the mechanism(s) related to the post-transcriptional or protein synthesis level. In general, a primary notion has been that the regulatory mechanisms must be relatively host independent to facilitate the interspecies transfer of genes coding for R-M systems. Any reliance on the specific cellular host factor may limit the spread of genes. On the other hand, the successful installation of genes requires an optimal fitting to host gene expression machinery including the recognition of promoter sequences by host RNA polymerase and refactoring to host codon usage. Sequence analysis of numerous R-M systems revealed genes that are different from host genes in features such as GC content, dinucleotide frequencies and codon usage^35^. This led us to focus on the codon usage for a few R-M systems isolated from *E. coli*. Of the systems evaluated, EcoVIII of *E. coli* E1585-68, turned out to be especially rich in rare codons for *E. coli*^36–38^.

We show that an abundance of rare codons are clustered within the EcoVIII REase gene and may seriously impact its translation. We hypothesise that the expression of the endonuclease is modulated by host genetic context, and we propose a novel, post-transcriptional mode of R–M system regulation that may help alleviate the lethality of unbalanced restriction enzyme gene expression.

## Material and Methods

### Bacterial strains and plasmids

The *E. coli* K-12 strains used in this study are described below. MC1061 [*araD139* Δ(*ara, leu*)7697, Δ*lacX74*, *galU*, *galK*, *hsdR*, *strA*] was used in *lacZ* reporter assays^39^. *E. coli* DH5α and MM294 were used for all other purposes including the cloning steps. *E. coli* Rosetta and BL21(DE3) were employed in gene overexpression^40,41^. MP060 and MP064 strains containing the yellow fluorescent protein (YFP) fused to the promoter of *sulA* (P_*sulA*_*-yfp*) was employed to test the SOS response^12^. The plasmids used are listed in Table 1. Plasmids constructed in this study were deposited in the Collection of Plasmids and Microorganisms, University of Gdansk, Poland.

**Table 1:** Plasmids used in this study and their relevant features.

Name	Relevant features/genotype	Reference
pRARE	pACYC184 derivative carrying genes for tRNAs of the rare codons of *E. coli*: *argU* (AGG,AGA), *ileX* (AUA), *leuW* (CUA), *proL* (CCC), *glyT* (GGA), Cm^R^	^40^
pLysS	pACYC184 derivative carrying gene of T7 lysozyme, a natural inhibitor of T7 RNA polymerase, Cm^R^	Novagene
pKRP10	chloramphenicol resistance cassette, Cm^R^	^99^
pEC156	natural plasmid carrying EcoVIII R-M system	^18, 37, 100^
pT7-6/pT7-3	inducible ϕ10 promoter of T7 phage, Ap^R^	^101^
pT7-EcoVIIIM	as pT7-6, but gene for EcoVIII MTase cloned under, ϕ10 promoter as transcriptional fusion with its own promoter	^36^
pT7-3cm	as pT7-3, but chloramphenicol resistance cassette introduced to break the bla gene, Cm^R^	This study
pRR	as pT7-3, EcoVIII R-M genes cloned under ϕ10 promoter,	This study
pFF	as pRR, but two codons of EcoVIII REase gene substituted as followed R(AGG)16→R(CGC); R(AGG)17→R(CGC)	This study
pRF	as pRR, but one codon of EcoVIII REase gene substituted R(AGG)17→R(CGC)	This study
pFR	as pRR, but one codon of EcoVIII REase gene substituted R(AGG)16→R(CGC)	This study
pRR0	as pRR, but EcoVIII REase gene inactivated (R−M+)	This study
pFFcm	as pFF (R + M+), but chloramphenicol resistance cassette introduced to break the bla gene, Cm^R^	This study
pFF0cm	as pFFcm, but EcoVIII REase gene inactivated (R−M+), Cm^R^	This study
pLex-3B	vector for testing gene translational fusion to *lacZ* reporter, pBR322 ori, promoter-less *lacZ* gene, Ap^R^	^43^
pLexRR	as pLex3B, but WT fragment encompassing the natural gene promoter, rbs and first 29 codons of EcoVIII REase gene fused in frame to *lacZ*, Ap^R^	This study
pIM-RM	pACYC184 derivative carrying genes of WT EcoRI R−M system, R + M+; Cm^R^	^28^
pIM27	as pIM-RM, but EcoRI REase gene inactivated, R−M+; Cm^R^	^28^
pIMEcoRI-FF	as pIM-RM, but WT rare codons of REase AGG_CUA at position 9 and 10 replaced by frequent ones CGC_CUG; R + M+; Cm^R^	This study

### T7 RNA polymerase dependent protein induction and pulse-chase assay

Single clones of BL21(DE3) [pLysS/pRARE] cells, which inducibly express T7 RNA polymerase and constitutively express T7 lysozyme (pLysS; Novagene) or genes encoding tRNAs for rare arginine codons AGA, AGG, and CGA, glycine codon GGA, isoleucine codon AUA, leucine codon CUA, and proline codon CCC (pRARE, Novagene), were grown overnight at 37 °C in M9 minimal medium^42^ supplemented with 0.2% glucose, ampicillin (100 μg/ml) and chloramphenicol (34 μg/ml). Cells were then diluted 100-fold in the same medium without glucose. When the cultures reached OD_600nm_ of 0.4, they were induced with 1 mM IPTG. Rifampicin (SIGMA) was used 30 min later at 200 μg/ml to block transcription by *E. coli* RNA polymerase. For labeling the synthesized proteins, 300 μl aliquots of cultures were collected at given times and incubated with 7 μCi/ml [^35^S]methionine (1.45 Ci/mmol, Amersham Corp.) for 20 min at 37 °C. Cells were then pelleted, washed and dissolved in SDS-PAGE lysis buffer (10 mM Tris-HCl pH 8.0, 10% sucrose, 1 mM EDTA, 2.5% SDS, 0.001% bromophenol blue). Proteins were run on a 10% SDS-PAGE, and the gels were dried, blotted and exposed to X-ray film (KODAK).

### Construction of *ecoVIIIR::lacZ* reporter and LacZ activity assay

The translational fusions of *ecoVIIIR* fragment carrying arginine codons fused to the *lacZ* reporter gene were generated as follows. The 280 bp of *ecoVIIIR* gene fragment containing the natural promoter and ribosome binding site was amplified by PCR (primers: GAGCTCGAGTTAAAGCGTGGG and CAGGATCCCCACTTAATTTGAC introducing XhoI and BamHI site, respectively), cleaved with XhoI and BamHI and cloned into a pLex3B vector^43^ that had been linearized with the same restriction enzymes. This yielded pLexRR (arginine codons WT- AGA AGA) in which *lacZ* is preceded by the *ecoVIIIR* promoter and 29 codons in the same reading frame. All constructs generated were confirmed by sequencing. The LacZ assays were based on hydrolysis of *o*-nitrophenyl-β-D-thiogalactoside (ONPG, SIGMA) using exponentially growing cells in M9-glucose minimal medium at 37 °C as described elsewhere^21,44^.

### Fluorescence assay

Cells were grown with shaking to exponential phase in LB medium^42^, gently pelleted, washed once with PBS buffer and then resuspended again in 500 µl of PBS buffer. Half of the sample was used to monitor the optical density (600 nm) of bacteria and the other half was used to read the YFP intensity (emission at 515 nm with an excitation at 545 nm) in a 96-well plate reader (EnSpire Multimode; Perkin Elmer). Relative fluorescence was corrected by subtracting the level of fluorescence of non-YFP bacterial cells and dividing by the optical density.

### Relative restriction activity assay

Restriction activity of *E. coli* cells carrying EcoVIII/EcoRI R–M system and its variants was measured using the plaque formation efficiency (EOP) of phage λ*vir*. There are 6 EcoVIII (HindIII) sites and 4 EcoRI sites in the λ*vir* genome. The EOP of λ*vir* was calculated as the ratio of plaques formed on *E. coli* MG1655 containing plasmid with no R-M system to those formed on the same strain containing a plasmid with the EcoVIII/EcoRI R–M system or their variants.

### Analysis of fitness effect by mixed culture competition experiments

Two comparably sized colonies of *E. coli* MG1655 picked from fresh transformation on LB-agar plates (with appropriate selective antibiotics) were inoculated into 5 mL of M9-glucose media. One colony carried the pRR plasmid with WT R–M system and was selected on ampicillin. The second colony was selected on chloramphenicol, and contained its variant – the pFFcm plasmid wherein two rare arginine codons at the *ecoVIIIR* gene were replaced by a higher frequency CGC codon. In addition, the *cat* gene was cloned to disrupt the *bla* gene of pFF to change the plasmid selection to chloramphenicol resistance. Control cultures were grown in parallel and contained cells with plasmids bearing restriction-negative and modification-positive variants (pRR0 vs. pFF0cm) or just empty vectors (pT7-3 vs. pT7-3 cm). These cultures were used to test whether the antibiotics-resistance genes alone give some advantage in a mixed cell population. At time zero of the competition experiment, 1:1 mixed cultures of competing cells were inoculated. Every 15–18 hrs of incubation at 37 °C with shaking, the co-cultures were diluted 10^6^ into fresh minimal media without antibiotics. Samples of each mixed competition culture were immediately diluted and then spread quantitatively onto either LB-agar containing the appropriate selective antibiotic or onto LB agar without antibiotics. The colonies were counted and the ratio of colony-forming units (CFU) from the two competing cell populations was calculated using T = (CFU_cm_/CFU_amp_); their generation number was also determined. Data were normalized using the results from the vector control [V = (CFU_cm_/CFU_amp_)]. The relative competitive fitness (W) was then calculated as W = log[T/V] for each tested generation time-point, as previously described^29^.

## Results

### The rare arginine codon clusters of the EcoVIII R–M system are features of a horizontally transferred cassette

We analyzed the DNA sequence, for the *E. coli* E1585-68: restriction endonuclease (REase) and DNA methyltransferase (MTase). We found a significant difference in the overall GC content of the R-M system unit (less than 38%) compared to its natural carrier - plasmid pEC156 (49.3%) and to the *E. coli* genome (50.8%). We also modelled the codon usage pattern for the EcoVIII R–M system genes using the codon adaptation index (CAI)^45,46^. Genes with low CAI indices reflect the optimal codon usage of their former host^35^. The CAI values for REase and MTase are 0,151 and 0,185 respectively^36^, and indicate that both belong to the group of *E. coli* genes that were most likely acquired by horizontal gene transfer. The results are consistent with data on codon usage deviation for other R–M systems isolated from *E. coli* (EcoRI and EcoRV) and from other members of the *Enterobacteriaceae* family (KpnI, SmaI, SinI, and PvuII)^35^.

**Figure 1:**
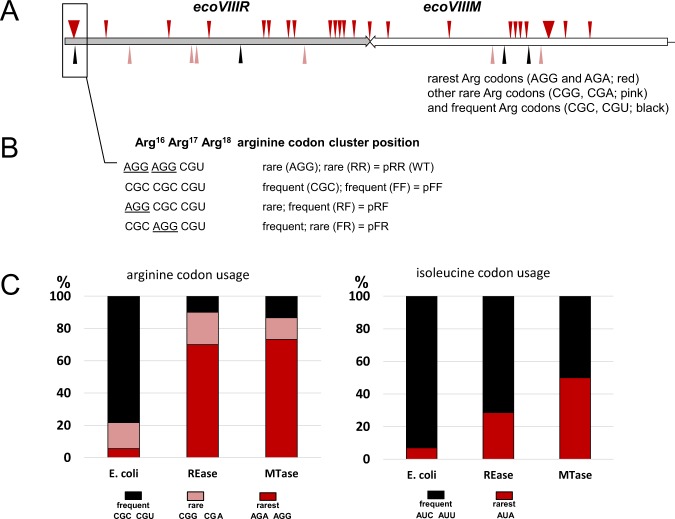
Representation of arginine codons in the EcoVIII REase and MTase genes. (**A**) Distribution of arginine (Arg) codons (in scale). The positions of rarest AGG and AGA Arg codons are indicated above gene arrows in red, whereas other rare CGG and CGA Arg codons are annotated below the genes, marked in pink. The most common Arg codons for *E. coli* are also marked below the genes, in black. Two Arg clusters are marked as thick triangles, others represent the single occurrences. (**B**) The position of cluster of rare Arg codons within the WT REase gene is denoted as RR in pRR plasmid (rare codon at 16^th^; rare codon at 17^th^). Codon variants were mutagenized to generate REase variants encoded as FR (frequent codon at 16^th^; rare codon at 17^th^); RF (rare codon at 16^th^; frequent codon at 17^th^) or FF (both frequent codons). At all gene variants the REase amino acid sequence remains unchanged. (**B**) Codon usage for arginine and leucine for *E. coli* genes compared to EcoVIII REase and MTase genes represented here as the percentage content of particular codon (frequent/rare/rarest) in entire pool of codons for analyzed amino-acid (Supplementary Table S1). *E. coli* values are based on 4332 coding sequences (1372057 codons) for *E. coli* K12 deposited at Codon Usage Database (www.kazusa.or.jp/codon/).

Our analysis of the *E. coli* codons in the EcoVIII R–M system revealed an overrepresentation of rare arginine (AGG and AGA) (>70%) and rare isoleucine (AUA) codons (Fig. 1A,C, Table S1). Out of the 20 arginine codons in the REase gene 18 are rare, and 13 of 15 are rare in the MTase gene. A high number of these rare codons cluster close to the C-terminus of REase, whereas the rare codons in the MTase remain in the central part of the gene (Fig. 1A). The rare isoleucine codons for MTase are closer to N-terminus, but distributed evenly across the REase. The arginine codons, however, form a cluster of two rare codons (tandem AGA) and one frequent codon starting at amino acid position 16 near the N-terminus of the REase. In case of MTase the AGG tandem is at position 167–168 (Fig. 1B). It has been shown that the presence of rare codon cluster close to the gene start may cause a translational complication^47^.

### Effects of rare arginine codon cluster on EcoVIII enzymes synthesis

**Figure 2:**
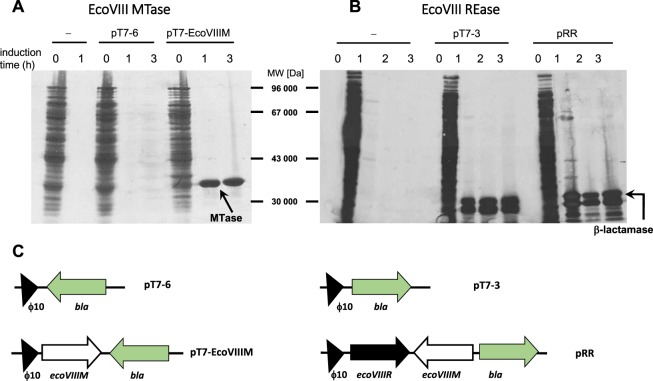
Effect of rare arginine codons on EcoVIII enzyme synthesis. *E. coli* BL21(DE3) pLysS cells containing a compatible plasmid from pT7 series carrying EcoVIII MTase gene (pT7-EcoVIIIM) (**A**) or EcoVIII REase gene (pRR) (**B**) both under T7 promoter, as well as their parallel controls, were grown until log phase, induced with 1 mM IPTG and labeled with [^35^S]-methionine at the post-induction time (one, two and three hours) as described under “Material and Methods”. Time point 0 represents a sample taken before induction. 200 μg/ml rifampicin was added to cultures 30 min after IPTG induction to block the host RNA polymerase and allow for exclusive expression from the T7 promoter. Time-point samples were taken and their proteins were resolved on 10% SDS-PAGE and visualized by autoradiography. Molecular weight markers are shown. Arrows indicate the labeled products: EcoVIII MTase (36 kDa) (**A**) and the precursor and mature form of β-lactamase (31.5 and 28.9 kDa) (**B**). A schematic representation of plasmid constructs used is shown in (**C**).

To test the effects of rare arginine codons on EcoVIII REase and MTase synthesis in *E. coli* cells, we made transcriptional fusions and used ϕ10 promoter of T7 phage. In this system, the T7 RNA polymerase gene is under the control of an IPTG inducible *lac*UV5 promoter that is located on *E. coli* BL21(DE3) chromosome^41^. In addition, we used the pLysS plasmid to provide a T7 lysozyme, which is a natural inhibitor of T7 RNA polymerase^48^, to decrease the basal expression of the toxic REase gene. EcoVIII MTase and REase synthesis was monitored using a pulse-chase assay carried out in minimal media in the presence of [^35^S]-methionine after IPTG induction and rifampicin addition to block *E. coli* RNA polymerase, but not T7 RNA polymerase (Fig. 2).

The induction of *ecoVIIIM* expression led to a prominent and exclusive MTase synthesis (36 kDa) (Fig. 2A). In contrast, EcoVIII REase synthesis was not observed under the same conditions (Fig. 2B). As a control, we used a plasmid, wherein the *ecoVIIIR* precedes the *bla* gene in the same orientation (Fig. 2C). Only β-lactamase (precursor and mature form) synthesis was detected, regardless of the presence or absence the preceding EcoVIII REase gene (Fig. 2B). We concluded that the observed translational defect may be due to the presence of the two consecutive rare arginine codons in the REase gene (AGG^16^AGG^17^), close to the translation initiation site (within 25 first codons). The presence of the AGG/AGA codons in this location may confer a severe effect on initiation of protein synthesis^49–52^. In addition, no tandem of rare codons was detected within the 25 first codons of *ecoVIIIM* gene, but there are single rare isoleucin codons (AUA) present at positions 11, 22, 25. It seems these codons do not cause any problem for MTase overproduction in this case (Fig. 2A). The rare arginine tandems have no severe effect when are present beyond the first 25 triplets^53^. We also found rare arginine codons located in the central part of the EcoVIII MTase gene (AGA^122^AGA^123^), but still observed that efficient MTase synthesis was achieved (Figs 1 and 2A).

### The presence of two consecutive rare arginine codons in *ecoVIIIR* affects translation efficiency

**Figure 3:**
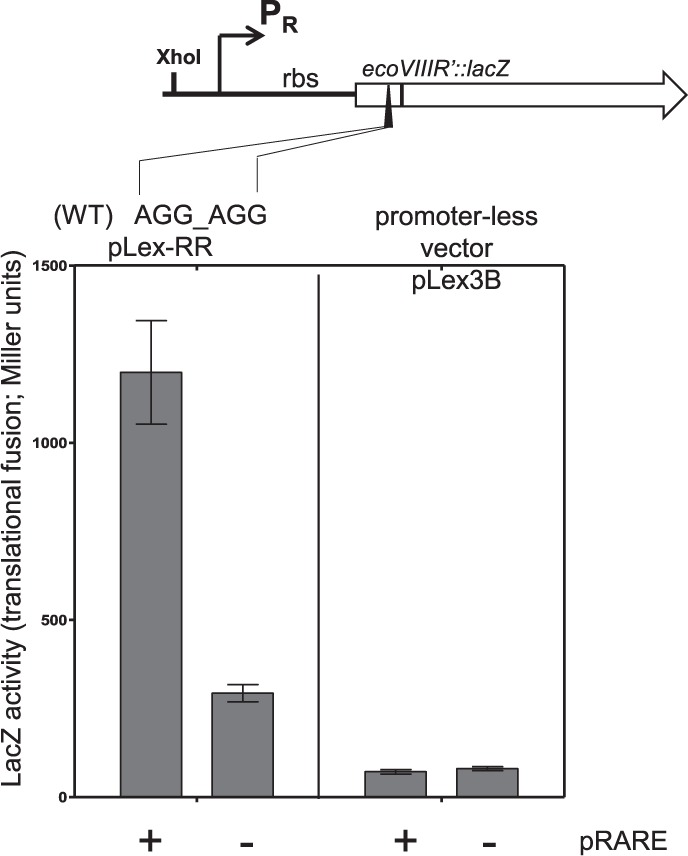
Cluster of rare arginine codons in REase gene blocks its translation. This effect is suppressed, when genes coding for rare tRNAs for *E. coli* are introduced into the cell by the pRARE plasmid. The expression of REase gene is measured by translational fusion to *lacZ* reporter assay presented on the scheme. The control plasmid (pLex3B) was used in parallel. Standard deviation from at least three measurements is indicated.

To test the effects of the tandem rare arginine codons on *ecoVIIIR* translation efficiency, we used a quantitative *lacZ* reporter assay. We generated translational fusions of WT *ecoVIIIR* with the promoter-less *lacZ* gene in the pLex-3B vector. The *lacZ* reporter gene in such construct was preceded by the natural promoter region, ribosome binding site and 29 N-terminal *ecoVIIIR* codons in the same reading frame (pLex-RR). The reporter assay shows the high LacZ activity was achieved only when the plasmid pRARE carrying the genes for tRNAs for the rare codons including tRNA^AGG/AGA^ was present (Fig. 3).

**Figure 4:**
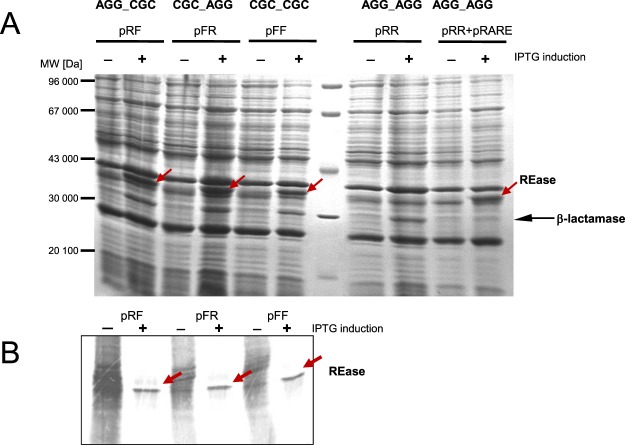
Synthesis of WT EcoVIII REase and its variants with rare codons substituted by high frequency codons for *E. coli*, as indicated on Fig. 1B. (**A**) *E. coli* BL21(DE3) cells containing a plasmid with REase variant were induced with 1 mM IPTG or not induced (control). Then, the samples were taken and proteins resolved on 10% SDS-PAGE and visualized by Coomassie staining. Molecular weight markers are shown. The arrows indicate the band corresponding to the REase product (37 kDa) obtained only if at least one rare codon was replaced (pRF; pFR; pFF) or pRARE plasmid carrying the genes for rare tRNAs was present (**B**). The exquisite REase production (indicated by an arrow) was also confirmed by the pulse chase assay, as described in Fig. 2. The full blot is presented at Figure S1 of the Supplementary Material.

To further investigate the effect of clustered arginine codons, we replaced the rare codons with higher frequency variants and measured whether REase synthesis is overcome after induction of *ecoVIIIR* gene expression from the T7 promoter. To do this, we constructed variants of the REase gene by site-directed mutagenesis, wherein each rare codon cluster (AGG, pRR) is replaced by a tandem of frequent codons (CGC; pFF/pRF/pFR; Fig. 1B). Next we performed overexpression experiments as shown before (Fig. 2). We compared the total protein content from cells with and without IPTG induction to determine if R.EcoVIII (36,7 kDa) was generated for each of the variants. We observed, that replacing a single rare arginine codon from the tandem is sufficient to unleash REase overproduction (compare pRR versus pFF; pRF and pFR induction positive lanes in Fig. 4A left panel).

Moreover, if *E. coli* cells with pRR plasmid carrying WT EcoVIII R–M system were supplemented with tRNA^Arg^ from the pRARE plasmid then inducible REase overproduction occurs indicating that the translational suppression is relieved (Fig. 4A right panel). To confirm these observations, we also performed a pulse-chase assay for cells carrying plasmids with the REase gene, having the rare arginine codons substituted. We used the same conditions as in the experiment shown in Fig. 2B. The experiments revealed the exclusive presence of a comparably intense band for the EcoVIII REase for each variant when at least one rare arginine codon was replaced (Fig. 4B). We also tested whether the REase mRNA stability is affected by the presence of genes for rare codons carried by pRARE plasmid. We used the approach, where the cultures of *E. coli* MG1655 carrying pRR plasmid with and without pRARE, were treated with the rifampicin, which inhibits the initiation of transcription. The results clearly show that no significant change in mRNA level for REase gene has been found. The determined half-life of REase mRNA was about 2 min. (Supplementary Fig. S2).

Overall, these results indicate that the inhibition of R.EcoVIII synthesis under overexpression conditions is primarily caused by the presence of tandem rare arginine codons at the N-terminus of the *ecoVIIIR* gene (positions 16 and 17).

### Substitution of the arginine tandem increases REase restriction activity and induces the cell filamentation

To further understand the role of the rare arginine cluster in EcoVIII R–M expression, we examined how their presence or absence affects restriction activity under steady-state levels, when gene expression signals come from a constitutive promoter and other natural genetic elements. We used λvir bacteriophage to compare plaque formation for the WT EcoVIII R–M system and the three variants with codon substitutions in the tandem arginine codons (Fig. 1B). The rare arginine codons (AGG_AGG; pRR, WT), when substituted with CGC_AGG (pFR); AGG_CGC (pRF) or CGC_CGC (pFF) conferred comparable and almost seven-fold higher restriction than the wild-type (Table 2). We also tested the activity of REase variants *in vitro* using crude cell extracts and substrate DNA to calculate the enzyme activity in units per amount of total cellular protein content. The results were similar to the *in vivo* observations (not shown) and taken together clearly indicate the biological significance of the rare arginine clusters present at the 5′ end of ecoVIIIR gene. We also supplemented the WT EcoVIII R–M system with pRARE carrying the genes for tRNA of rare codons (arginine, leucine, isoleucine, glycine, proline; Table 1) and observed significantly higher levels of restriction (38-fold) (Table 2). In this case, we concluded, although the rare arginine codon cluster seems to be important for the initiation of translation, however the rare arginine codons outside the cluster, as well as other rare codons, such as isoleucine and glycine present in a high number throughout both REase and MTase genes also affect their expression (Fig. 1A, Supplementary Table S1).

**Table 2:** Substitution of rare arginine codons in REase gene increases restriction activity of EcoVIII R–M system.

Plasmid	Genotype	Plaque-forming units	^a^Efficiencyof plaque formation	Restriction relative to WT (R + M+)
pT7-3	R^−^ M^−^ (vector)	(4.5 ± 0.02) × 10^6^	1	
pRR0	R^−^ M^+^	(4.3 ± 0.02) × 10^6^	0.9	
pRR	R^+^ M^+^(WT) AGG_AGG^b^	(3.5 ± 0.04) × 10^5^	0.078	1
pFR	R^+^ M^+^ CGC_AGG^b^	(5.3 ± 0.02) × 10^4^	0.012	6.5
pRF	R^+^ M^+^ AGG_CGC^b^	(4.9 ± 0.02) × 10^4^	0.011	7.1
pFF	R^+^ M^+^ CGC_CGC^b^	(4.4 ± 0.03) × 10^4^	0.0097	8.0
pT7-3; pACYC184		(4.5 ± 0.03) × 10^6^	1	
pT7-3; pRARE		(3.7 ± 0.04) × 10^6^	0.82	
pRR; pACYC184		(3.4 ± 0.04) × 10^5^	0.075	1
pRR; pRARE		(8.9 ± 0.03) × 10^3^	0.002	38

R, restriction; M, modification; WT, wild-type.

^a^Efficiency of plaque formation = plaque-forming units on strains carrying tested plasmid divided by plaque-forming units on pT7-3 vector.

^b^Arg codons at position 16 and 17 of *ecoVIIIR*.

All plasmids having R^+^ M^+^ genotype carry R–M system with intact amino acid sequence for EcoVIII REase and MTase. The host bacterium was *E. coli* MG1655. Standard deviation from four measurements is indicated.

**Figure 5:**
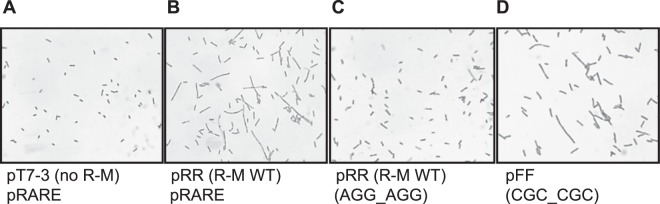
Microscopic images for morphology analysis of *E. coli* MG1655 cells carrying plasmids without **(A)** and with EcoVIII R-M system of WT (**C**) or its variant with two rare arginine codons replaced by high frequency codons, as exemplified by the pFF plasmid (**D**). Cells with two compatible plasmids of: WT (pRR) and pRARE (**B**). Cell filamentation appears if REase expression is upregulated - (**B**) and (**D**).

We examined the cell morphology for the R–M system variants and the microscopic images revealed the presence of cell filamentation, a typical manifestation of SOS response induction, for the variants with at least one rare codon replaced with a higher frequency variant (pRF, pFR or pFF), but not for WT R–M system alone (pRR) (Fig. 5C vs. D). However, when the plasmid carrying the WT EcoVIII R–M system was supplemented with pRARE plasmid

extensive cell filamentation occurred (Fig. 5B vs. A,C). These data are in agreement with the restriction activity test, as the elevated restriction of phage DNA correlated with SOS response induction.

### Lack of rare arginine codon cluster in REase gene impairs the cell fitness

**Figure 6:**
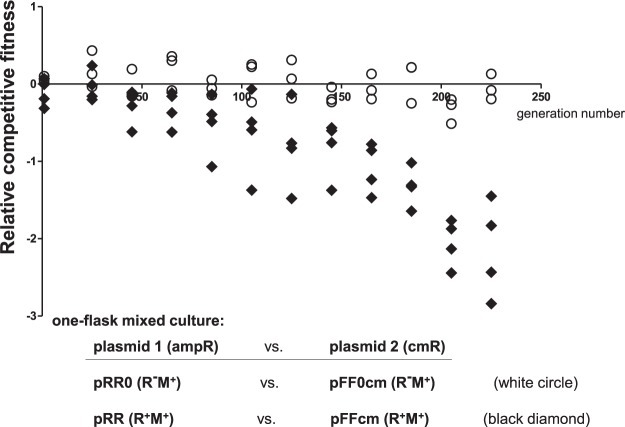
Relative fitness of cells with a variant of EcoVIII R-M system with high frequency codon cluster at its REase is heavily impaired. Mixed cultures were prepared by adding equal number of two types of competing *E. coli* cells into medium without any antibiotic (Material and Methods). Each type carried a plasmid with a specific R-M system variant and its distinct antibiotic marker (chloramphenicol or ampicillin), as indicated below the diagram. One flask co-cultures were diluted every 24 generations into fresh media and CFUs of competing cells were measured. Relative competitive fitness (W) was estimated individually for each mixed culture represented on the diagram as a single symbol, calculated as W = log(CFU_cm_/CFU_amp_) and normalized to vector control (pT7-3 vs. pT7-3cm) (Materials and Methods). Black diamonds represent four separate co-cultures, where the WT R-M system in ampicillin resistant cells (pRR) competed with its R-M system variant with frequent codon cluster in REase gene (pFFcm) in chloramphenicol resistant cells. In control, parallel co-cultures of cells with plasmids with restriction-negative and modification-positive variants (pRR0 vs. pFF0cm; white circles) were used.

We also questioned whether or not the WT EcoVIII REase or its variant with cluster of arginine codons replaced by high frequency codons for *E. coli*, would show any differences in viability or fitness under long-term culture conditions. New plasmid constructs were prepared carrying the R-M system variants or vectors control, where the chloramphenicol resistance cassette was inserted into *bla* gene in order to restore the ampicillin sensitivity (pT7-3cm - vector, pFF0cm – R^−^M^+^ and pFFcm – R^+^M^+^) making antibiotic resistance the cell marker to monitor the numbers of competing strains over the entire growth course. For the competitions, cells carrying the appropriate plasmids (pRR WT, R^+^M^+^ ampR vs. pFFcm, R^+^M^+^, cmR) were mixed 1:1 and inoculated into the same flask (with replicates). The cultures were prepared in minimal media and kept over 220 generations without the antibiotic pressure with sub-culturing every 20/21 generations (CFU were counted at these time points). For controls, parallel flasks containing the restriction negative variants (pRR0, R^−^M^+^ ampR vs. pFF0cm; R^−^M^+^ cmR) and compatible vectors to normalize the data (pT7-3; R^−^M^−^ ampR vs. pT7-3cm; R^−^M^−^ cmR) were grown under the same conditions. We observed a significant and continuous loss of cells with the frequent codon clusters (pFFcm) when cultured with the strain bearing the WT version of the rare codon cluster (pRR) indicating a loss of fitness that likely due to higher REase levels (Fig. 6).

Moreover, we tested also the presence of restriction activity for cells derived from the last counting (about 220 generation) and found that some fraction of cells from pFFcm pool, but not pRR, had changed their initial highly-restrictive phenotype into restriction-negative. We isolated these plasmids and observed some changes using fragmentation of plasmid DNA with various restriction enzymes. For the control co-cultures with restriction-negative variants, a difference in relative fitness was not detected (pRR0 vs pFF0cm). The number of the competing cells stayed comparable for the entire course of the experiment (Fig. 6).

### Rare codon clusters at other R–M system units

To determine if what we observed in the EcoVIII R-M system occurs in the other Type II systems, we surveyed the REBASE data, for “true” (not putative) Type II R-M systems, whose genes are cloned, sequenced and isolated from an *E. coli* host^54^. Next, we analyzed the codons for the REase and MTase genes to determine if the rare codon clusters are also formed at the N-terminus of other similar REases. There are single rare codons in several R-M systems, but out of 22 R-M systems surveyed, we found only two examples, where a cluster of rare codons was detected: EcoRI and Eco57I/Eco9272I. These codons were at positon of 9 and10, and coded for different amino-acids (arginine and leucine) in both cases: AGG_CUA (Table 3). In most cases, one to three rarest codons within 25 first codons were detected at REase gene, and comparable number at MTase gene (Table 3).

**Table 3:** Distribution of rarest codons near start of translation for Type II R-M systems isolated from *E. coli* strains.

Type II R-M system	Position of rare codons within first 25 codons of gene coding for:	
	REase	MTase
EcoVIII	AGGAGG (16,17)	no
EcoRI	AGGCUA (9,10)	AGA (3)
Eco128I	CUA (10)	NA
EcoRII	CUA (8)	no
EcoT38I	AUA (15)	GGG (12,15)
Eco1524I	GGA (21)	no
Eco29kI	AGA (8,14)	AGA (2)
	CUA (19)	CUA (17)
	AUA (22)	GGA (9,21)
EcoGIII	AUA (15)	CUA (5)
	AGA (22)	
EcoO109I	AUA (7)	GGG (15)
	AGA (21)	
	CUA (25)	
EcoRV	CUA (11)	AUA (10)
		AGA (25)
EcoHK31I	AGA (12)	no
Eco47II	no	no
EcoGIII	AUA (15)	CUA (5)
	CCC (21)	CCC (17)
	AGA (22)	
Eco31I	GGA (4)	M1 AUA (4)
		M2 no
	**REase/MTase fused into one polypeptide**	
Eco4465II	no	
Eco57I/Eco9272I	AGACUA (9,10)	
Eco644I/Eco933II	no	
EcoE1140I	CUA (25)	
EcoMVII	no	
EcoPT54I	no	
EcoTWII	no	
Eco9276II	CUA (25)	

Data based on available sequence source of REBASE collection on Nov. 2018^54^. Rarest codons are: AGA/AGG – arginine; CUA – leucine; AUA – isoleucine; GGA – glycine; CCC – proline. NA – sequence not available.

**Figure 7:**
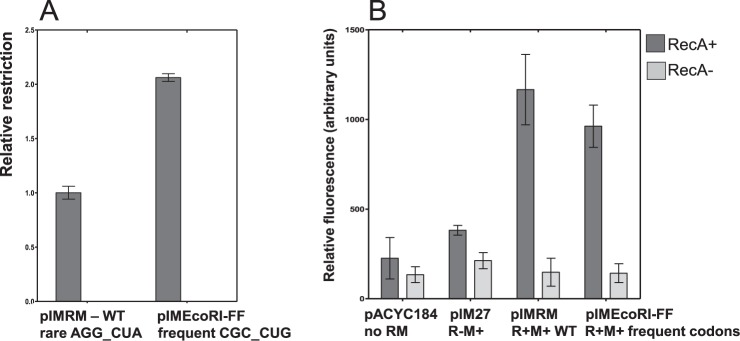
Effect of cluster of rare codons on REase from EcoRI R-M system. For WT and variant of EcoRI R-M system with frequent codons (FF) relative restriction as measured (**A**) and the SOS response induction in the context of *recA-* and *recA* + cells (**B**) was assayed. Standard deviation from at least three measurements is indicated.

Finally, we used the well-studied EcoRI R-M system as a test to see if changing of the rare codon cluster would significantly alter the gene expression and restriction activity in a manner similar to EcoVIII R-M system. We changed the rare codons AGG_CUA (pIM-RM) into the more frequent CGC_CUG (pIMEcoRI-FF) via site-directed mutagenesis. When compared to WT, the relative restriction level for the EcoRI R-M variant system (pIMRM vs. pIMEcoRIFF) are indeed higher (phage assay, Fig. 7A). However, the two-fold increase over WT is modest compared to the change observed for the EcoVIII R-M system (8-fold, Table 2) using the same phage assay with a comparable number of restriction sites on phage genome. This may be explained by the fact the EcoRI R-M system has cluster of two codons (arginine and leucine), that are delivered by two different tRNAs, and this affects translation of REase gene to lesser extent than in EcoVIII case. In addition, we also tested if the elevated restriction activity of EcoRI REase variant can induce the SOS response quantitativly using a *E. coli* YFP reporter strain (P_*sulA*_*-yfp*). The results show that the altered EcoRI R-M system is comparable to the parental (WT) in inducing some level of SOS signal (Fig. 7B).

## Discussion

Horizontal gene transfer is a major force driving the microbial evolution resulting in genome plasticity and mosaic pattern of their genes^55–58^. In general, the genes to be acquired need to successfully overcome the wide range of barriers to sustain the viability of the accepting host and to be retained in the genome^59,60^. The newly acquired DNA fragments with genes carrying the beneficiary function for the host have the highest chance to be integrated into the genome^61^. R-M systems certainly deliver the advantageous efficient anti-phage tools to their host^5^, but also once taken, remain utterly linked to the host as the addictive modules^62^. Losing the R-M system has the serious death consequences by mechanism of the post-segregational killing also typical for toxin-antitoxin systems^23^. EcoVIII R-M genes, carried by a natural ColE1-type plasmid, displayed the features of the horizontally transferred genes^36,37^ by conferring the atypical codon usage and biased GC content^63^. It is known that codon usage reflects an adaptation of genes to the translational machinery of their host, unlike the most recently acquired, alien genes^64^. It also seems that the successful lateral gene transfer is most likely to occur if the codon usage of entering genes is compatible with the accepting genome^65–67^. Moreover, if accepted, the genes also tend to adapt to the host tRNA pool for their better expression to maintain their function^68^. However, this process may not occur for certain genes, as for tested here EcoVIII R-M system, which maintained the unusual overrepresentation of rare codons for *E. coli*. Both EcoVIII R-M genes display a strong codon bias, which seems to be favored by the host because there is a strong selective pressure to keep the rare codons and provide an adequately low amount of protein to perform its function satisfactorily, for fitness and to minimize the R-M system toxicity, as reported by others^65,69,70^. Many rare codon cluster positions are conserved within homologous coding sequences across diverse bacteria, suggesting they result from a positive selection and have a functional role^71^. In particular, we tested the role of rare arginine cluster near the start codon of REase and found its potential regulatory function in protein synthesis, R-M system maintenance and impact on the host cell fitness. We found that the cluster of rare arginine codon alone negatively affects the REase synthesis, as the delivery of tRNAs for the rare arginine codons highly increased its expression, in accord with other reports related to production of heterologous proteins^72,73^. Indeed, the tRNAs’ availability seems to be the limiting factor for the protein synthesis, when the rare codon cluster is located close to translational start of the gene. We tested whether such cluster affects EcoVIII REase (positions 16 and 17) and MTase (positions 122 and 123) synthesis by pulse chase assay with a T7 promoter system. Our results confirmed the cluster position is detrimental for REase synthesis due to its proximity to translational start, but not for MTase, whose synthesis occurred efficiently. The REase translational suppression could be relieved in two ways: if more tRNA^Arg^ was delivered or at least one rare arginine codon within the cluster was replaced by a high frequency codon. The data from the REase overproduction in T7 promoter system matched well the natural promoter context, where we measured the level of relative restriction, which is the function of two active enzymes: REase and MTase. We also show that if only one rare arginine codon was replaced for the high frequency codon, the relative restriction due to REase synthesis was significantly augmented (by 7-fold). Such exchange seems to be deleterious to cell fitness. Using direct competition fitness assay, we show that the cells carrying the REase gene variant with cluster of high frequency arginine codons were outcompeted by the cells with WT REase. Outcompeted cells with elevated relative restriction (about 7-fold; REase with CGC cluster) were eliminated from co-cultures due to possible autorestriction of its host genomes. Clearly, loss of rare arginine codon cluster in REase gene also shifted the balance in REase/MTase activities and subsequently triggered the SOS response, manifested in cell filamentation. This phenomenon is frequently observed whenever R-M system balance is lost^12–14,74,75^.

There are many reports that link the position of translationally non-optimal codons near the start of the gene with modulation of its gene expression at the translational stage^76–79^. The presence of consecutive rare codons may lead to translational pausing, frameshifting and/or amino-acid misincorporation in the growing polypeptide chain due to delay in appropriate tRNA delivery^47,49–52,80,81^. The local pauses in translation may be beneficial for some protein synthesis and co-translational folding^82–85^. In particular, the NGG codons located near the initiation codon (+2 – +5 of codons) have a severe reducing effect on gene expression, but if shifted slightly downstream, the expression proceeds at normal level^86^. However, some reports also indicate that the effect of rare codons’ cluster is related rather to the mRNA secondary structure and its stability, and sometimes it may even enhance gene expression^87–90^. Genome-wide analysis also supports this observation, i.e. the relaxed mRNA structure at the beginning of gene is favored, not the codon usage^91^ and the non-optimal codons tend to destabilize mRNA^92^. When we made the RNA structure prediction for different arginine cluster variants using the RNAfold software, the result showed comparable structures (Supplementary Fig. S3).

The regulatory role of rare codon cluster at 5′ gene termini has been shown for bacteriophage lambda integrase^93^. In addition, even a single synonymous change for rare codon for certain genes could exert a strong phenotypic effect. Such a change at the *hfq* gene, a global RNA regulator of *S. typhimurium*, caused only a two-fold decrease of *hfq* expression, but showed a distinct phenotype of the strain virulence with reduced biofilm formation, motility and survival in macrophages^94^. For some genes, the presence of several rare codons in regulatory genes is required to keep their low expression and sustain the optimal operon coordination, like for FimB recombinase^95^ or global regulator BldA in *Streptomyces*^96,97^. These schemes of regulation rely on the accessibility of tRNAs for rare codons as a limiting factor for gene expression, as it also seems true for the reported here EcoVIII restriction endonuclease, whose upregulation may be toxic for the host strain. We screened more Type II R-M systems isolated from *E. coli* to find whether the rare codon-dependent regulation may be a more general phenomenon. We found several examples of REase genes with only a single rare codon present near the 5′ terminus, on average. In two cases, the cluster of two rare codons for different amino-acids was detected, for the EcoRI and Eco57I R-M system. We tested the effect of a synonymous change for the EcoRI REase, and found the relative restriction changed only by two-fold, much less than for the EcoVIII R-M system (7-fold).

For mobilizable R-M genes with toxic potency (also toxin and antitoxin systems), the flexible level of expression should be avoided, thus such a wide repertoire of regulatory mechanisms exist, where REase and MTase expression is controlled at the transcription step^23^. However, in this report, we indicate a novel aspect, never raised before, which is associated with translation of the REase. Its expression seems to be dependent on the pool of host tRNAs for arginine, which may be limiting in some bacteria, such as in *E. coli*, or not at all in bacteria which use the AGG codon frequently. In the latter case, the installation and optimal maintenance of EcoVIII R-M system may be problematic. During bacterial growth the tRNAs’ level also slightly changes^98^, so the R-M system is challenged to adapt to such conditions and not to kill its host. The example of the Type II EcoVIII R–M system shows the potency of the toxic gene expression, the restriction endonuclease, to be tuned to the host’s genetic context, that may help to alleviate the lethality of unbalanced R-M system gene expression.

## Supplementary information

Supplementary Info
