# Supplementary material for: Natural tuning of restriction endonuclease synthesis by cluster of rare arginine codons
Source: Sci Rep. 2019 Apr 9;9:5808. doi: 10.1038/s41598-019-42311-w (PMC6456624; doi:10.1038/s41598-019-42311-w)
Supplement: Supplementary file 1 — Supplementary Info [file 41598_2019_42311_MOESM1_ESM.docx]

**Natural tuning of restriction endonuclease synthesis**

**by cluster of rare arginine codons.**

**Supplementary Information**

##### **Iwona Mruk^1^*, Tadeusz Kaczorowski^1,2^ and Agata Witczak^1^**

^1^ Department of Microbiology, Faculty of Biology, University of Gdansk, Wita Stwosza 59, Gdansk 80-308, Poland

^2^ Laboratory of Extremophiles Biology, Department of Microbiology, Faculty of Biology, University of Gdańsk, Wita Stwosza 59, Gdansk 80-308, Poland

*Corresponding author

**SUPPLEMENTARY TABLE S1.** Representation of the rarest codons for *E. coli*, EcoVIII and EcoRI R-M system genes.

| **amino-acid** | **codon** | **Percentage content of particular codon**  **in entire pool of codons for analyzed amino-acid** | | | | |
| --- | --- | --- | --- | --- | --- | --- |
|  |  | **all *E. coli* genes *** | ***ecoVIIIR*** | ***ecoVIIIM*** | ***ecoRIR*** | ***ecoRIM*** |
| Arginine | **CGG**  **CGA**  **CGU**  **CGC** | **9.8** | **5.0** | **0.0** | **0.0** | **0.0** |
|  |  | **6.3** | **15.0** | **13.4** | **14.3** | **7.7** |
|  |  | **38.0** | **5.0** | **0.0** | **7.2** | **0.0** |
|  |  | **40.3** | **5.0** | **13.4** | **0.0** | **7.7** |
|  | **AGG**  **AGA** | **2.0** | **40.0** | **20.0** | **35.7** | **7.7** |
|  |  | **3.6** | **30.0** | **53.2** | **42.8** | **76.9** |
| Leucine | **CUG** | **49.9** | **11.6** | **0.0** | **0.0** | **10.7** |
|  | **CUA** | **3.6** | **4.0** | **17.4** | **16.7** | **17.8** |
|  | **CUU** | **10.3** | **15.3** | **8.7** | **20.8** | **17.8** |
|  | **CUC** | **10.4** | **11.6** | **8.7** | **12.5** | **3.6** |
|  | **UUG** | **12.8** | **23.0** | **8.7** | **12.5** | **17.8** |
|  | **UUA** | **13.0** | **34.5** | **56.5** | **37.5** | **32.3** |
| Isoleucine | **AUA**  **AUU** | **7.0** | **28.6** | **50.0** | **48.0** | **30.4** |
|  |  | **50.8** | **67.8** | **42.3** | **32.0** | **60.9** |
|  | **AUC** | **42.2** | **3.6** | **7.7** | **20.0** | **8.7** |
| Proline | **CCG** | **52.8** | **0.0** | **0.0** | **0.0** | **0.0** |
|  | **CCA** | **19.0** | **0.75** | **43.75** | **16.7** | **45.5** |
|  | **CCU** | **15.8** | **0.25** | **50.00** | **83.3** | **54.5** |
|  | **CCC** | **12.4** | **0.0** | **6.25** | **0.0** | **0.0** |
| Glycine | **GGG** | **15.0** | **14.3** | **8.0** | **31.6** | **29.4** |
|  | **GGA** | **10.8** | **14.3** | **32.0** | **21.0** | **17.6** |
|  | **GGU** | **33.6** | **71.4** | **40.0** | **42.1** | **35.3** |
|  | **GGC** | **40.6** | **0.0** | **20.0** | **5.3** | **17.6** |

*based on 4332 coding sequences (1372057 codons) for *E. coli* K12 W3110 deposited at Codon Usage Database (www.kazusa.or.jp/codon/).

Rarest codons are indicated by red font.

**SUPPLEMENTARY FIGURE S1.** Synthesis of EcoVIII REase variants with rare codons substituted by high frequency codons for *E. coli* by the pulse chase assay*.* The exquisite REase production (37 kDa) obtained only if at least one rare codon was replaced within the cluster (pRF; pFR; pFF). Expanded blot picture of **FIGURE 4B** of the main manuscript.

control control

pRF pFR pFF

**− + − + − + − + − +** IPTG induction


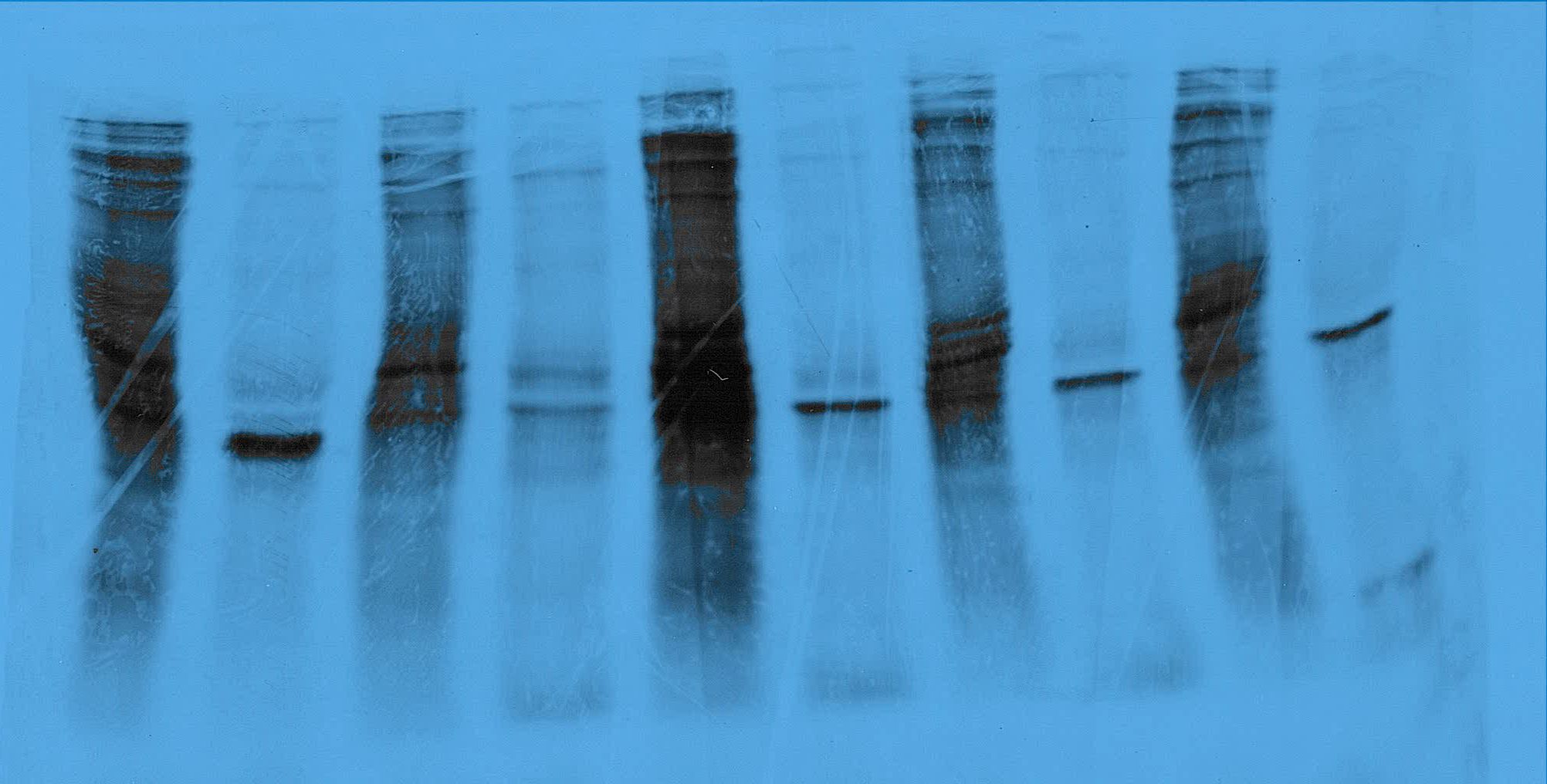


**SUPPLEMENTARY FIGURE S2.** EcoVIII REase transcript stability in the context of pRARE plasmid.

At time 0 the rifampicin (300 µg/ml) was added to an exponential cultures of *E. coli* MG1655 carrying pRR plasmid with and without compatible pRARE plasmid growing in LB medium. The time-point samples were collected and their RNA was isolated and cDNAs were prepared. The mRNA was determined via q-RT-PCR, using stable 5S rRNA as the internal standard.

Methods:

RNA extraction and cDNA synthesis

Collected time-point samples were immediately mixed with 1 ml of RNA Later (Sigma) and total RNA was isolated using the Total RNA Mini Plus Concentrator kit (A&A Biotechnology, Poland). The cDNAs were obtained, after RNase-free DNase I (Eurx, Poland) treatment, by using random hexamers and RevertAid First Strand cDNA Synthesis Kit (Thermo Scientific).

QRT-PCR

Two sets of primers were used to estimate REase transcript levels in qPCR reaction (Roche Light Cycler), specific to REase gene (R1 5’-GCAATGGATGGTTGGAGGAATA-3’ and R2 5’-GACTTGATTTAACTGGGAGCTGAT-3’) and specific to the reference gene 5S rRNA used as the internal, stable marker (5Sf 5’-ATGCCGAACTCAGAAGTGAAAC-3’ and 5Sr 5’-AGTTCCCTACTCTCGCATGG-3’). Each qPCR reaction (10 μl) contained 5 μl SG qPCR Master Mix (2×) with SYBR Green I fluorescent dye, Perpetual Taq DNA polymerase and dNTPs (Eurx Poland), 3 µl H_2_O, 1 μl of 5 μM forward and reverse primers mix and 1 μl of diluted cDNA as a template. The qPCR cycling conditions were as follows: pre-denaturation step in 94 ^o^C for 3 min and 35 cycles of 94 ^o^C for 20 s, anealing 60 ^o^C for 15 s and extension at 72 ^o^C for 20 s. Each reaction was performed in biological triplicates and repeated at least twice independently. Melting curve analysis was used to confirm the formation of the specific products.

**SUPPLEMENTARY FIGURE S3.** Predicted secondary structures for the 5’-end of mRNA for WT EcoVIII REase and its variants with rare arginine codons substituted.

The structures were generated by RNAfold (http://rna.tbi.univie.ac.at/cgi-bin/RNAWebSuite/RNAfold.cgi).

The values of a minimum free energy is indicated. The blue arrow shows the position of rare arginine codons at WT or the substitutions in the variants.
